# Collective intermittent exploration in fish schools is mediated by visual cues

**DOI:** 10.1098/rsos.250033

**Published:** 2025-06-25

**Authors:** Deze Liu, Daniel Burbano

**Affiliations:** ^1^ Electrical and Computer Engineering, Rutgers, The State University of New Jersey, Piscataway, NJ, USA

**Keywords:** animal movements, collective behaviour, stochastic differential equations

## Abstract

Exploratory behaviour is fundamental to animal collectives, directly influencing fitness through resource acquisition and predator avoidance. Despite its ecological significance, the processes governing when and how animals initiate exploration in social contexts under varying sensory inputs remain poorly understood. Here, we investigate how the number of individuals and illumination (modulating visual input) influence zebrafish exploratory behaviour. Visual cues were quantified via opacity (field occupancy) and optic flow (relative motion). With visual input, zebrafish exhibited higher swimming activity and shorter exploratory bursts, while its absence led to more dispersed and prolonged exploration. Interestingly, fish triads without visual input exhibited longer exploration times compared to dyads. A data-driven stochastic model revealed a bistable potential landscape balancing social cohesion and exploration, modulated by a parabolic noise term driving decision-making. Visual cues biased the potential towards conspecific proximity, while their absence diminished this effect, promoting prolonged exploration. The noise term follows an entropy-like pattern analogous to a coin flip, reaching maximal uncertainty at intermediate distances and forcing individuals to break indecision between proximity and exploration. These findings point to a complex interplay between sensory input and group dynamics, underscoring the profound impact of environmental changes and the number of conspecifics on collective behaviour.

## 1. Introduction

The emergence of collective behaviour in living groups is a pervasive phenomenon across the natural world, spanning multiple scales from cells [1] and insect swarms [2] to fish schools [3] and human crowds [4]. Behaviours such as swarming, schooling, flocking and herding arise from complex multisensory feedback processes, integrating local interactions with neighbours and environmental cues. Collective dynamics in animal groups have a long evolutionary history, conferring critical advantages such as enhanced predator avoidance, improved foraging efficiency and reduced locomotion costs [5]. In fish, for example, group living confers critical benefits [6–9], including enhanced predator avoidance through coordinated vigilance [10], energy conservation via hydrodynamic advantages, such as reduced drag and Kármán gaiting [11,12], and enhanced exploration [13]. In fact, exploratory behaviour in fish is often a socially facilitated process whereby individuals benefit from observing and interacting with conspecifics to navigate their environment, locate resources and assess potential risks [13–15].

For instance, in [14], the authors studied how predation pressure influences collective exploration in guppies (*Poecilia reticulata*) by comparing populations from habitats with differing levels of predation risk. It was established that guppies from high-predation environments exhibited reduced exploration, increased group cohesion and greater consistency in leadership roles, underscoring the role of predation in shaping collective dynamics and individual contributions to group decision-making. Similarly, in [13], the author investigated how the number of individuals and chemical cues modulate exploratory behaviour in juvenile mosquitofish (*Gambusia holbrooki*) within a novel environment. Larger groups displayed enhanced exploration and swimming activity, consistent with social facilitation, but this required direct visual contact, as chemical cues alone were insufficient. In [15], the authors studied how group size and flow velocity influence collective exploratory behaviour in juvenile Italian riffle dace (*Telestes muticellus*). Flume experiments revealed that exploration and swimming activity increased with the number of individuals and that this effect was amplified at higher flow velocities. The authors suggested that mechanisms to explain this behaviour included competition for favourable areas, stress reduction and energy savings from coordinated swimming.

Vision is a fundamental sensory modality across taxa [16], which has its evolutionary roots in aquatic environments, a legacy reflected in the shared anatomical structure of vertebrate eyes [17]. Fish, descendants of some of the earliest sighted vertebrates, such as haikouichthys [18], offer invaluable insights into the evolution of vision and its role in shaping collective behaviour. Vision in fish is believed to be the primary sensory modality modulating collective behaviour [19–23] when compared to other sensory modalities such as the acoustico-lateralis system and olfaction [24,25]. A recent empirical study on fish schools (*Hemigrammus rhodostomus*) [23] found that vision is essential for cohesive group behaviour, as fish failed to form schools at low light levels. Increasing illuminance led to a transition from disordered movement to polarization and stable rotational milling, with collective properties saturating above a threshold.

Although vision has been found to be a crucial sensory modality driving collective behaviour [19–23], the interplay between the number of individuals and visual cues in shaping collective fish exploration remains largely unexplored, leaving fundamental questions unanswered. For instance, how do fish decide when and how to initiate exploration in a social context, particularly under fluctuating visual cues caused by changing illumination? What dynamics govern these decision-making processes, and how are they influenced by social and illumination factors? This gap in knowledge limits our understanding of the principles driving complex collective behaviours, particularly given that exploration is critical in dynamic and/or unfamiliar environments, as it is an essential behaviour for survival and ecological success [13–15].

Here, we sought to fill this gap by integrating zebrafish (*Danio rerio*) experiments with data-driven modelling of stochastic dynamic systems, offering a quantitative framework to explore the interplay of visual input, number of individuals and collective decision-making in driving exploratory behaviour. Specifically, we analysed how the Number of Individuals (Singles, Dyads and Triads) and Illumination (Bright and Dark, which modulates visual input) influence fish exploratory behaviour. Adult zebrafish served as our model biological system, chosen for their rich social behaviours and suitability for studying collective phenomena [26,27]. In the wild, zebrafish typically inhabit turbid waters, where light levels vary (i) seasonally due to factors such as rainfall, sediment levels and water flow, and (ii) spatially based on depth, vegetation cover and local water clarity [28]. This ecological context makes studying their responses to fluctuating illumination particularly relevant, as their natural environment exposes them to dynamic light conditions. Zebrafish, a tropical freshwater species, has become a cornerstone of scientific research across diverse disciplines [29], ranging from translational neuroscience [30] to biomedical studies, enabling advancements in understanding human diseases, drug discovery and therapeutic evaluation [30,31]. Due to their unique advantages ranging from a fully sequenced genome [32] to detailed characterization of their brain function [33], zebrafish enable the possibility of investigating the neural and genetic basis of behaviour [34]. Moreover, the selection of the Number of Individuals (Dyads and Triads) was informed by the rich interaction dynamics observed in fish dyads [35,36] and recent evidence that zebrafish triads could exhibit many phenomena characteristic of larger groups [27], making them an excellent choice for studying collective exploratory behaviour.

We conducted experiments with zebrafish in a shallow, novel environment under two illumination conditions: infrared light, invisible to adult zebrafish (Dark) and standard visible light (Bright). Swimming activity and exploration were quantified using standard metrics, including linear and angular speeds and interindividual distance. Visual input was assessed through two salient metrics: opacity, which measures the occupancy of the zebrafish’s visual field, and optic flow, which captures the pattern of apparent motion within the visual field caused by the relative movement between the fish and their surroundings [37]. Building on our previous work on zebrafish dyads [36] and studies demonstrating that reduced swimming activity in guppies and rummy-nose tetras is influenced by turbidity [38] and varying illumination [23], we hypothesize that (i) zebrafish will exhibit higher swimming activity in the presence of visual cues compared to their absence, with this effect modulated by the number of individuals. (ii) Visual input, quantified by opacity and optic flow, will shape the temporal and spatial dynamics of exploration, with the magnitude of this modulation varying as a function of the number of individuals. Additionally, we established a systematic framework to infer and model fish exploration. This approach resulted in a computational model based on stochastic differential equations [39], providing a quantitative approach to understanding the mechanisms underlying collective behaviour as a function of visual input.

## 2. Methods

### 2.1. Experimental setup

**Figure 1.**
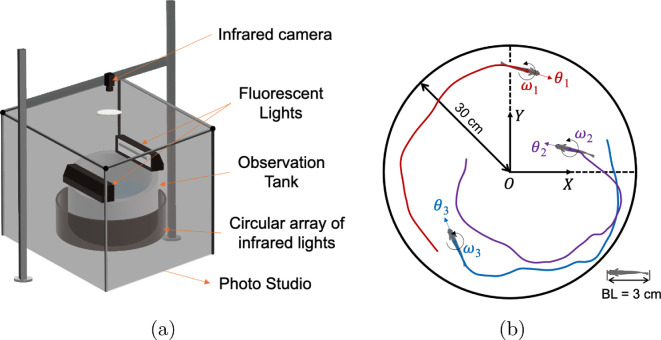
Illustration of the experimental setup. (a) Overview of the experimental apparatus. (b) Coordinate system for overhead-tracked fish swimming trajectories within a circular tank, illustrating centroid positions, heading angle and angular speed relative to the global Cartesian reference frame with origin O and axes X and Y.

The experimental setup illustrated in figure 1a consisted of a circular tank of 60 cm in diameter enclosed by opaque curtains (black photo studio) to provide homogenous lighting and also help eliminate external disturbances. The setup included two illumination configurations for video recording fish behaviour. *Bright condition*: two fluorescent lights (Aqueon Single Tube Strip Light, Central Aquatics Division of Central Garden & Pet Company, Franklin, Wisconsin, USA) were mounted above the tank to provide uniform lighting. *Dark condition*: three 940 nm infrared LED strips (360DigitalSignage 940 nm Waterproof Light Strips, Shenzhen Huake Light Electronics CO., LTD., Shenzhen, Guangdong, China) were arranged on a circular plastic sheet, forming a homogenous ring of illumination around the arena. The 940 nm wavelength was selected because it falls outside the spectral sensitivity range of adult zebrafish [40]. To video record fish behaviour in Bright and Dark, we utilized an infrared camera (FLIR Grasshopper3 GS3-U3-41C6NIR-C, Teledyne FLIR, Wilsonville, Oregon, USA) placed above the tank. The water depth was maintained at 10 cm during all experiments to promote predominantly horizontal motion [41,42].

### 2.2. Animal care and maintenance

A total of 120 wild-type zebrafish (*Danio rerio*) were utilized in this study. Fish were randomly selected from a larger population of 240 individuals, consisting of 44% females and 56% males, with an average body length of 3 cm. The animals were obtained from the zebrafish facility at Rutgers Robert Wood Johnson Medical School and housed in two 135-gallon vivaria (182.88 cm (length) × 60.96 cm (width) × 45.72 cm (height)). To ensure optimal water quality, the vivarium was equipped with two Fluval FX6 canister filters (Fluval, Hagen Inc., Montreal, Quebec, Canada). Fish were kept in one holding tank to be acclimated for approximately four months prior to the beginning of the experiment. The second vivaria was utilized to hold fish after being utilized in the experiment. The tank’s temperature was consistently maintained at 28${,}^{\circ }$C and the pH level at 7 through regular monitoring. Fish underwent a 12-hour light/12-hour dark photoperiod, with lights turning on at 08.00. Fish were fed tetra tropical flake food (TetraMin, Spectrum Brands Pet LLC, Blacksburg, Virginia, USA) once a day at approximately 19.00. Standard tap water, conditioned with the appropriate amount of API Stress Coat + water conditioner (API Stress Coat+, Mars Fishcare North America Inc., Chalfont, Pennsylvania, USA), was used to maintain water quality.

### 2.3. Experimental conditions and procedure

We investigated fish collective behaviour as a function of two independent variables; namely, Number of Individuals and Illumination. Number of Individuals was categorized as Singles, Dyads and Triads, while Illumination had two conditions, Bright and Dark, corresponding to visible light and non-visible infrared light, respectively. We conducted experiments consisting of six experimental conditions: Singles-Bright, Singles-Dark, Dyads-Bright, Dyads-Dark, Triads-Bright and Triads-Dark. Each condition consisted of 10 trials, resulting in a total of 60 trials. All fish were experimentally naive and were used only once. To account for potential time-of-day effects, these trials were randomly distributed across 5 days, with 12 trials conducted each day. Experimental sessions were consistently scheduled between 09.00 and 16.00. For each trial, we first hand-netted a fish from the holding tank and transferred it to the experimental arena. Then, we allowed the fish to acclimate to its new environment for a period of 10 min. This time was selected based on our previous work [43]. After the habituation period, we video-recorded fish behaviour for 10 min under the specified experimental condition. Upon completion of the trial, the fish was transferred to a separate holding tank. After completing all the experiments, we observed that two video files were corrupted and removed from the final dataset. One trial was from condition Dyads-Bright, and another trial was from Triads-Bright.

### 2.4. Fish tracking

All videos for each trial were recorded at 30 frames s^−1^, resulting in a total of 18 000 frames per video. Fish swimming trajectories were extracted using the open-source tracking software AnimalTA (v. 2.3.1) [44]. This software features automated tools for maintaining individual fish identities across frames by resolving occlusions, along with manual editing capabilities to improve data accuracy. Before initiating tracking, we set the pixel-to-centimetre ratio to 34.13 pixels cm^−1^ and adjusted contrast parameters to enhance target (fish) differentiation from the background. After completing the automatic tracking process, we conducted a manual review of all fish trajectories to verify identity consistency and resolve complex occlusions that could not be addressed automatically. The software output generated time series data representing the two-dimensional position $\left(\{x(\tau ){\}}_{\tau =1}^{\ell },\{y(\tau ){\}}_{\tau =1}^{\ell }\right)$ with a total of ℓ=18000 samples. These positions were measured in centimetres relative to the centre of the experimental tank, as illustrated in figure 1b.

### 2.5. Quantifying swimming activity, visual cues and exploratory behaviour

We utilized two-dimensional position data to quantify fish swimming activity through the computation of linear and angular speeds. Specifically, linear speed was computed through $v(\tau )=\sqrt{v_{x}^{2}(\tau )+v_{y}^{2}(\tau )}$, where $v_{x}(\tau )$ and $v_{y}(\tau )$ are the components of the velocity vector along the horizontal (X) and vertical (Y) axes, respectively. These components were determined through numerical differentiation using the first-order forward difference method. Absolute angular speed was computed as the absolute value of the angular speed given by


(2.1) $$\omega (\tau )=\frac{1}{\delta_{t}}\text{atan2}(|[\text{p}(\tau ),\text{p}(\tau +1)]|,\text{p}(\tau )\cdot \text{p}(\tau +1)),$$

where the time increment is $\delta_{t}=1/30$, |⋅| denotes the determinant and ⋅ represents the dot product. The vectors p(τ) and p(τ+1) indicate the fish position at times τ and τ+1, respectively.

**Figure 2.**
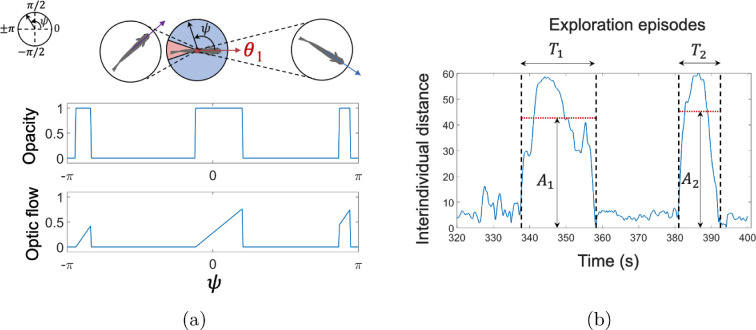
Quantifying visual cues and intermittent exploratory behaviour. (a) Schematic representation of opacity and optic flow quantification for a focal fish with heading angle $\theta_{1}$ and visual field represented by visual rays ψ. The red region indicates the blind visual angle of the fish. (b) Example of an empirical time series of interindividual distance, illustrating intermittent exploration episodes characterized by durations $T_{1}$ and $T_{2}$, with amplitudes $A_{1}$ and $A_{2}$, defined as the median (red dashed line) of the interindividual distance over the respective time intervals.


Visual input was quantified through two salient metrics; namely, Opacity and Optic flow. Opacity quantifies the occupancy of the zebrafish’s visual field [37], while Optic flow represents the pattern of apparent motion within the visual field caused by relative movement between the fish and its surroundings [45]. Specifically, Opacity, O takes values in the interval [0,1] where O=0 indicates no visual obstructions in the visual field, while O=1 indicates the entire visual field is covered figure 2a. This metric was computed as [37]

(2.2) $$\begin{array}{lcr} & O=\frac{1}{2\pi -\gamma }\int_{-\pi }^{\pi }V(\psi )\;1_{A}\;d\psi . & \end{array}$$

Here, the function V(ψ) is a piecewise constant function represented as a set of unit boxcar functions (or shades) that denote the visual field of a fish. Specifically, V(ψ)=1 if the visual ray at an angle ψ (measured relative to the fish’s local frame) intersects with the perimeter of a conspecific; otherwise, V(ψ)=0. The fish perimeter was assumed to be a circle with a diameter of 1 BL =3 cm. Function $1_{A}$ with A={ψ∉(−γ/2,γ/2)}, is the indicator function that takes values of $1_{A}(\phi )=1$ if ϕ∈A and $1_{A}(\phi )=0$; otherwise. The parameter γ represents the blind angle, determined based on the findings of [46], which reported that the zebrafish blind area spans $21^{\circ }\pm 2.30^{\circ }$.

Optic flow, denoted by F, quantifies the relative motion detected within a zebrafish’s visual field and is defined as


(2.3) $$\begin{array}{lrr} & F=\int_{-\pi }^{\pi }|W(\psi )|\;1_{A}\;d\psi , & \end{array}$$

where W(ψ) represents the angular speed of objects across various visual rays, measured at angle ψ. This metric was computed based on the angular displacements observed at two consecutive time steps (see electronic supplementary material, Section 1).

Fish exploratory behaviour was quantified by calculating the time-averaged interindividual distance, defined as the Euclidean distance between fish. For triads, three pairwise interindividual distances were computed, and their average was used to represent the overall interindividual distance for the group. Additionally, intermittent exploration was characterized by analysing the duration and amplitude of exploration episodes. Specifically, the duration was calculated from time series data of interindividual distances by identifying intervals when a focal fish, after being in close proximity, suddenly moved to a distance greater than 3 BL from others and maintained that separation for over 3 s. Amplitude was computed as the average interindividual distance over each exploration episode (figure 2b). For each trial, these durations and amplitudes of exploration episodes were averaged to obtain the duration and amplitude of exploration episodes.

### 2.6. Data-driven mathematical modelling

Consider a stochastic differential equation of the form [39]


(2.4) $$\begin{array}{lrr} & dy(t)=F(y(t))\;dt+G(y(t))\;dW(t), & \end{array}$$

where, y(t)∈ℝ is the state of the system at time t, F:ℝ↦ℝ is the drift term, G:ℝ↦ℝ is the diffusion term and W(t) is a standard Wiener process. The primary goal of data-driven modelling is to accurately estimate the drift and diffusion terms in equation (2.4) from time series data of y(t). To do so, we establish the following pipeline based on three main steps as follows.

*Step 1*: *Filtering data:* We first filter empirical data of y(t) by using a fourth-order Butterworth low-pass filter with a cutoff frequency of $\omega_{c}=0.1$ rad s^−1^ using Matlab (v. R2023a). Next, we compute the time derivative of y(t) using the forward difference method.

*Step 2*: *Estimating drift and diffusion:* Using the filtered data, we estimate the F(y) and G(y) using the Kramers–Moyal formulae [47,48] via


(2.5)F^(y(t))=limδt→0[1δt(y(t+δt)−y(t))],(2.6)G^(y(t))=limδt→0[1δt(y(t+δt)−y(t))2],

where $\delta_{t}$ is a small time increment. The output of this estimation step consists of time series of the estimates $\{\hat{F}(\tau ){\}}_{\tau =1}^{\ell -1}$ and $\{\hat{G}(\tau ){\}}_{\tau =1}^{\ell -1}$.

*Step 3*: Finally, we establish the functional form of F(y) and G(y) by formulating and solving a Stepwise Sparse Regression (SSR) problem [47]. Specifically, we consider the following optimization problem


(2.7) $$\begin{array}{lrr} & \hat{\Xi }=\arg\;\min_{\Xi }\|Y-\Theta (y)\;\Xi {\|}_{2}^{2}+\lambda {\left\|\Xi \right\|}_{1}, & \end{array}$$

where $Y\in {\mathbb{R}}^{\ell -1}$ represents the observations, that is, either the time series $\{\hat{F}(\tau ){\}}_{\tau =1}^{\ell -1}$ or $\{\hat{G}(\tau ){\}}_{\tau =1}^{\ell -1}$ obtained in step 2. $\Theta \in {\mathbb{R}}^{\ell -1\times p}$ is the design matrix or (library matrix) constructed from polynomial terms of the independent variable from degree 0 up to degree p−1. λ is the regularization parameter enforcing sparsity. Y is the independent variable. Ξ is the vector of coefficients determining the active terms of the nonlinear function of drift or diffusion. We used LASSO regression, implemented via the coordinate descent method [49,50], to initialize the coefficient matrix Ξ. This estimate was refined using a Sequential Sparse Regression (SSR) algorithm, which iteratively removes coefficients in Ξ with absolute values below a predefined threshold to enforce sparsity. The reduced set of predictors was then reintroduced into a LASSO regression to obtain re-estimated coefficients. The performance of the final model was evaluated using K-fold cross-validation, where the predictive error δ was computed as:


(2.8) $$\begin{array}{lcr} & \delta =\frac{1}{K}\sum_{k=1}^{K}\left(\frac{1}{n_{k}}\sum_{i=1}^{n_{k}}{\left(Y_{i}^{\left(k\right)}-{\hat{Y}}_{i}^{\left(k\right)}\right)}^{2}\right), & \end{array}$$

with K-fold cross-validation, the dataset is split into K subsets, with one-fold serving as the test set and the remaining K−1 folds as the training set. $n_{k}$ denotes the number of samples, $Y_{i}^{\left(k\right)}$ the observed value and ${\hat{Y}}_{i}^{\left(k\right)}$ its prediction. The process terminates when the mean squared error (MSE) from cross-validation ceases to decrease.

### 2.7. Statistical analysis

We used a two-way analysis of variance (ANOVA) to evaluate the effects of Number of Individuals and Illumination on linear and angular speeds, as well as on the duration and amplitude of exploration episodes. The swimming activity metrics were treated as the response variables, while Number of Individuals and Illumination were considered as independent variables. To simultaneously examine the impact of Number of Individuals and Illumination on visual cues and exploratory behaviour, we utilized a multivariate analysis of variance (MANOVA) with interindividual distance and opacity/optic flow as the response variables and Number of Individuals and Illumination as independent variables. Linear regression analyses were further conducted to explore the relationship between interindividual distance and visual cues (opacity/optic flow). Post hoc analysis was conducted using Tukey’s Honest Significant Difference (HSD) test to identify significant differences across the levels of Number of Individuals and Illumination conditions. All statistical analyses were conducted in R (v. 4.3.2). The significance level was set at 0.050.

## 3. Results

### 3.1. Linear and angular speeds vary as a function of illumination

We first investigated standard kinematic metrics, such as linear and angular speeds, to evaluate zebrafish swimming behaviour as a function of Illumination and Number of Individuals. These metrics quantify fish locomotory activity, with linear speed capturing the overall rate of forward movement. In contrast, absolute angular speed measures the statistical dispersion of fish turns, with higher values indicating greater variability or erraticity in turning behaviour [51,52]. These metrics provide a proxy of swimming activity and indicate potential changes in behaviour or navigational strategies [43].

**Figure 3.**
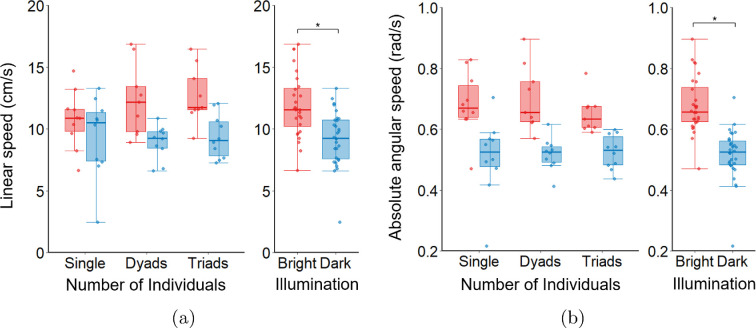
Analysis of the effect of Illumination and Number of Individuals on fish swimming activity, represented by (a) linear speed and (b) angular speed. Each box plot displays the complete dataset (light blue/red points), the median (solid red/blue line), the interquartile range (blue/red box indicating the first and third quartiles) and the data range excluding outliers (blue/red whiskers). Red colour and blue colours denote Bright and Dark conditions, respectively. Statistical significance (p<0.050) is denoted by the symbol ∗. Sample size: N=58 trials in total (approx. 10 per condition), excluding two corrupted files, one from Dyads-Bright and one from Triads-Bright.

Our analysis revealed that, compared to the absence of visual cues in Dark, zebrafish exhibit significantly higher linear speed (main effect of Illumination: $F_{1,52}=18.367;p<0.001;\eta^{2}=0.261$; figure 3a) and angular speed (main effect of Illumination: $F_{1,52}=46.780;p<0.001;\eta^{2}=0.474$; figure 3b) in Bright. In addition, we found that linear (main effect of Number of Individuals: $F_{2,52}=0.690;p=0.506;\eta^{2}=0.026$; Illumination × Number of Individuals: $F_{2,52}=1.059;p=0.354;\eta^{2}=0.039$; figure 3a) and angular (main effect of Number of Individuals: $F_{2,52}=0.248;p=0.781;\eta^{2}=0.009$; Illumination × Number of Individuals: $F_{2,52}=0.598;p=0.554;\eta^{2}=0.022$; figure 3b) speeds were unrelated to the number of conspecifics. These results indicate that, regardless of the Number of Individuals, fish tend to engage in higher locomotor activity in the presence of visual cues, while this is significantly diminished in the absence of them.

### 3.2. Intermittent exploration is modulated by visual feedback

We next explore the effect of visual feedback on exploratory behaviour by analysing the interindividual distance and visual cues metrics of opacity and optic flow as a function of Illumination and the Number of Individuals.

**Figure 4.**
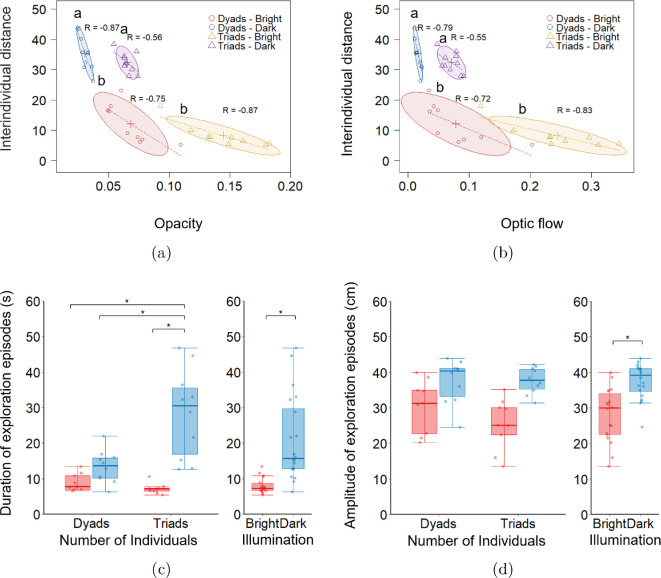
Analysis of the effect of Illumination and Number of Individuals on exploratory behaviour. (a) Opacity and interindividual distance. (b) Optic flow and interindividual distance. Each ellipse represents the confidence region of the bivariate normal distribution for the relationship between the interindividual distance and opacity/optic flow under combinations of visual feedback and Number of Individuals. Different letters indicate statistical differences across both dependent variables. Dashed lines represent linear regression fits, with R indicating the correlation coefficient. Analysis of intermittent exploratory behaviour quantified by the (c) duration and (d) amplitude of exploration episodes. Each box plot displays the complete dataset (light blue/red points), the median (solid red/blue line), the interquartile range (blue/red box indicating the first and third quartiles) and the data range excluding outliers (blue/red whiskers). Red colour and blue colours denote Bright and Dark conditions, respectively. Statistical significance (p<0.050) is denoted by the symbol ∗. Sample size: N=38 trials in total (approx. 10 per condition), excluding two corrupted files, one from Dyads-Bright and one from Triads-Bright.

Multivariate analysis of variance revealed that fish maintain larger interindividual distances in Dark with low values of opacity compared to the Bright condition, where opacity is high (main effect of Illumination: $F_{2,33}=105.068;p<0.001;Pillai=0.864$; figure 4a). Fish exhibit a similar effect on interindividual distance and optic flow (main effect of Illumination: $F_{2,33}=103.685;p<0.001;Pillai=0.863$; figure 4b). We registered that these differences are modulated by the Number of Individuals for both cases, that is, interidividual distance and opacity (main effect of Number of Individuals: $F_{2,33}=58.502;p<0.001;Pillai=0.780$; Illumination × Number of Individuals: $F_{2,33}=10.070;p<0.001;Pillai=0.379$; figure 4a) and interidividual distance and optic flow (main effect of Number of Individuals: $F_{2,33}=31.409;p<0.001;Pillai=0.656$; Illumination × Number of Individuals: $F_{2,33}=10.494;p<0.001;Pillai=0.389$; figure 4b). Post hoc analysis revealed that fish maintained a larger interindividual distance in the Dark compared to Bright for either fish dyads (p<0.050) and triads (p<0.050). However, no significant differences in interindividual distances were registered between dyads and triads under either illumination conditions. In addition, fish swimming in pairs exhibited lower opacity values compared to those in triads under both Bright (p<0.050) and Dark (p<0.050) illumination. Similarly, optic flow values were lower for dyads than for triads in both Bright (p<0.050) and Dark (p<0.050) conditions.

Covariance ellipse representations, combined with linear regression analysis, revealed a significant negative correlation between interindividual distance and visual cues. Under Bright conditions, fish dyads exhibited a strong negative relationship between interindividual distance and opacity (slope=−254.045; R=−0.750; p<0.050; $r^{2}=0.505$; figure 4a). Similarly, triads displayed a negative relationship (slope =−118.938; R=−0.870; p<0.010; $r^{2}=0.715$; figure 4a). Comparable trends were observed for the relationship between interindividual distance and optic flow for fish dyads (slope =−83.948; R=−0.720; p<0.050; $r^{2}=0.453$; figure 4b) and triads (slope =−47.423; R=−0.830; p<0.010; $r^{2}=0.649$; figure 4b), respectively. In Dark, the relationship between interindividual distance and visual cues was more sensitive, with higher slopes indicating that small input variations produced large output variations. This sensitivity limits the ability of visual metrics to explain interindividual distance and, hence, exploratory behaviour. Specifically, a negative relationship was registered between interindividual distance and opacity in dyads (slope =−1128.977; R=−0.870; p<0.010; $r^{2}=0.720$; figure 4a) and in triads (slope =−328.264; R=−0.560; p=0.094; $r^{2}=0.225$; figure 4a). A similar relationship was registered for optic flow in fish dyads (slope =−946.981; R=−0.790; p<0.050; $r^{2}=0.576$; figure 4b) and triads (slope =−129.890; R=−0.560; p=0.010; $r^{2}=0.215$; figure 4b).

Finally, we assessed intermittent exploration by analysing the duration and amplitude of exploration episodes as a function of Illumination and Number of Individuals. Our results revealed that Bright illumination promotes shorter bursts of exploratory behaviour compared to Dark (main effect of Illumination: $F_{1,34}=31.976;p<0.010;\eta^{2}=0.485$; figure 4c), and that these differences are modulated by the Number of Individuals (main effect of Number of Individuals: $F_{1,34}=9.744;p<0.010;\eta^{2}=0.223$; Illumination × Number of Individuals: $F_{1,34}=13.837;\;p<0.010;\;\eta^{2}=0.289$; figure 4c). Specifically, post hoc analysis revealed that fish triads spent significantly more time engaging in far-distance exploration under Dark compared to Bright (p<0.050) and both dyads under Dark (p<0.050) and Bright (p<0.050). In addition, we registered that in the absence of visual cues, fish increases the amplitude of exploration episodes, indicating a tendency to maximize their interindividual distances compared to Bright (main effect of Illumination: $F_{1,34}=22.364;\;p<0.010;\;\eta^{2}=0.397$; figure 4d). This result was found to be independent of Number of Individuals (main effect of Number of Individuals: $F_{1,34}=1.312;\;p=0.260;\;\eta^{2}=0.037$; Illumination × Number of Individuals: $F_{1,34}=0.753;\;p=0.194;\;\eta^{2}=0.049$; figure 4d).

### 3.3. Data-driven modelling suggests the presence of bistable dynamics mediated by visual cues

**Figure 5.**
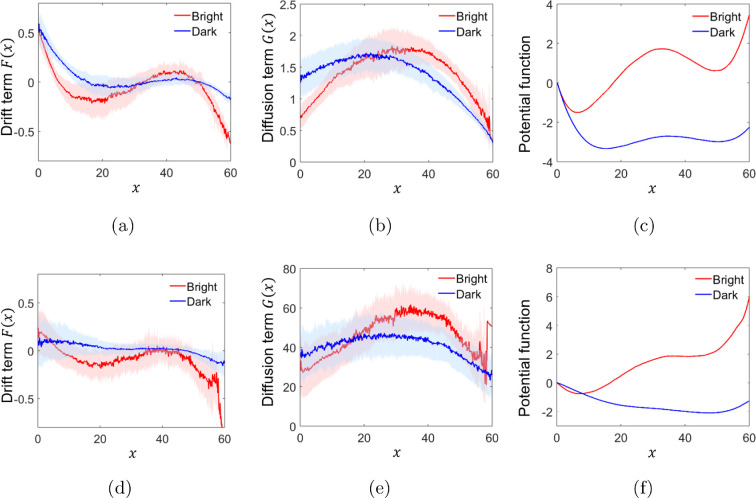
Dynamics of fish exploratory behaviour estimated from experimental data. Panels (a–c) present the drift term, diffusion term and potential function estimated for fish dyads, while panels (d–f) display the corresponding terms for fish triads. The solid red line represents the mean estimate across trials under bright illumination, and the solid blue line represents the mean estimate under dark illumination. Shaded regions in light red and blue colours denote the standard deviation.

Given our focus on analysing fish exploratory dynamics, we selected interindividual distance, denoted as x(t) (cm), as the primary dynamic variable to model. Within the existing literature on modelling fish behaviour, stochastic processes are commonly utilized to capture the variability inherent in undulatory swimming movements and the unpredictability of decision-making [53,54]. Gautrais *et al.* [53] introduced a persistent turning walker model [54], characterized by a first-order stochastic differential equation, to describe the angular velocity of *Kuhlia mugil*. Subsequent studies have extended this framework to describe fish behaviour in both open and confined environments [41,42], as well as in more complex scenarios such as swimming under flow conditions [55] or analysing predator–prey interactions [56]. Building on these works, we propose to capture the time evolution of interindividual distance x(t) using a stochastic differential equation of the form 


(3.1) $$\begin{array}{r} dx(t)=F(x(t))\;dt+G(x(t))\;dW(t),\;x(0)=x_{0}, \end{array}$$

where the function F(x) is the drift term encapsulating the deterministic component of the system dynamics. G(x) is known as the diffusion term, modulating the influence of the stochastic component driven by W(t), a standard Wiener process. By utilizing the system identification method outlined in §2.6, we estimated the drift and diffusion terms for each trial under varying Illumination and the Number of Individuals. The results are shown in figure 5a,b and figure 5d,e for fish dyads and triads, respectively. Averages across trials were calculated by binning the data into intervals of 0.1 cm. Notably, our identification process of the interindividual distance (see electronic supplementary material, Section 3) indicates that the drift follows a cubic relationship, while the diffusion term exhibits a parabolic dependence, that is $F(x)=a_{3}x^{3}+a_{2}x^{2}+a_{1}x+a_{0}$ and $G(x)=b_{2}x^{2}+b_{1}x+b_{0}$, respectively. By numerically integrating the average trajectory of the drift term using the trapezoidal rule, we obtained an estimated function that exhibits the characteristics of a potential function as shown in figure 5c–f. Note that under Bright illumination, both fish dyads and triads display a biased potential function, facilitating transitions between staying close to conspecifics and engaging in exploratory behaviour.

Based on these insights, we propose that the drift term can be modeled as F(x(t))=−∂V(x(t))/∂x(t) with V(x(t)) being a potential function of the form


(3.2) $$\begin{array}{lrr} & V(x(t))=-\alpha \;x(t)-\beta {\left(x(t)-x_{l}\right)}^{2}{\left(x(t)-x_{h}\right)}^{2}. & \end{array}$$

Here, the constant parameter β (cm${,}^{-2}$s${,}^{-1}$) determines the height of the barrier between the potential wells, while $x_{l}$ (cm) denotes the well minimum corresponding to the average interindividual distance during close-proximity swimming, and $x_{h}$ (cm) represents the well maximum associated with exploratory behaviour. The parameter α (cm s^−1^) encapsulates the bias of the potential function, where α=0 indicates an unbiased potential while larger values of α indicate a stronger preference for close-proximity swimming with conspecifics (electronic supplementary material, figure 2).

Moreover, note that the parabolic diffusion term amplifies noise at intermediate interindividual distances and minimizes it near the wells. While its shape remains consistent across Bright and Dark conditions, with only a slight central shift, the diffusion term exhibits higher amplitude in triads compared to dyads, indicating greater noise influence in triadic decision-making.

To the best of our knowledge, existing models [41,42,53,54,57–60] do not explicitly account for the bistable dynamics of exploratory behaviour and differentiate between conditions with and without visual input. Our modelling results complement previous efforts on fish modelling by demonstrating that exploratory behaviour is governed by a nonlinear, noise-driven potential function modulated by sensory cues. Our findings call for more refined mathematical models that can describe complex emergent behaviours as a function of sensory input.

### 3.4. Model validation via *in silico* experimentation

**Figure 6.**
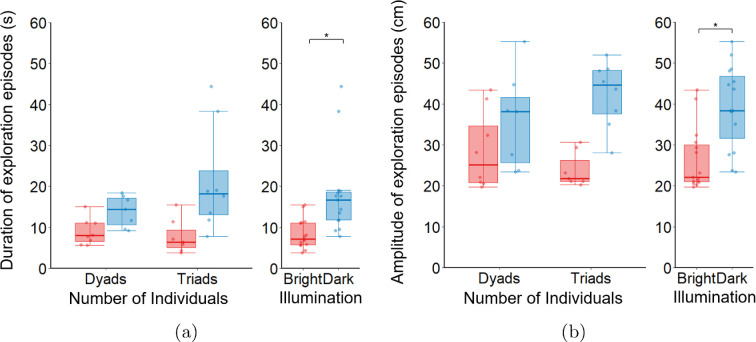
Analysis of *in silico* experiments on (a) duration and (b) amplitude of exploration episodes. Each box plot displays the complete dataset (light blue/red points), the median (solid red/blue line), the interquartile range (blue/red box indicating the first and third quartiles) and the data range excluding outliers (blue/red whiskers). Red colour and blue colour denote Bright and Dark conditions, respectively. Statistical significance (p<0.050) is denoted by the symbol ∗. Sample size: N=30 trials in total (approx. 10 per condition), excluding two outliers for Dyads-Bright and Triads-Dark and three from Dyads-Dark and Triads-Bright.

The model validation process consisted of replicating the distributions and statistical properties of the duration and amplitude of exploration episodes observed in the empirical data. To do so, we numerically integrated the model in (3.1) with F(x)=−∂V(x)/∂x, V(x) given by (3.2), and $G(x)=b_{2}x^{2}+b_{1}x+b_{0}$. Integration was performed using the Euler–Maruyama algorithm using the calibrated parameters from empirical data (see Section 3 in the electronic supplementary materials for more details). To maintain consistency with the experimental design, the same number of trials was conducted for each condition: Dyads-Bright, Dyads-Dark, Triads-Bright and Triads-Dark. The simulated results are shown in figure 6. Notably, *in silico* results captured the distributions observed from empirical data (figure 4c). While the Number of Individuals did not exhibit statistically significant differences, the main effects on Illuminations were successfully replicated. Specifically, after identifying and excluding outliers using the interquartile range rule, we found that fish under Bright illumination have shorter bursts of exploratory behaviour compared to Dark (main effect of Illumination: $F_{1,26}=11.689;p<0.050;\eta^{2}=0.310$; figure 6a). This result was found to be independent of the Number of Individuals (main effect of Number of Individuals: $F_{1,26}=1.927;p=0.177;\eta^{2}=0.069$; Illumination × Number of Individuals: $F_{1,26}=2.499;p=0.126;\eta^{2}=0.088$; figure 6a). In the absence of visual cues, the amplitude of exploration episodes increased compared to Bright (main effect of Illumination: $F_{1,26}=15.923;p<0.010;\eta^{2}=0.380$; figure 6b). This result was found to be independent of Number of Individuals (main effect of Number of Individuals: $F_{1,26}=0.307;p=0.584;\eta^{2}=0.012$; Illumination × Number of Individuals: $F_{1,26}=2.979;p=0.744;\eta^{2}=0.096$; figure 6b).

## 4. Discussion

Our results indicate that zebrafish exploration is strongly influenced by both visual cues and the number of individuals. In the presence of visual input, fish exhibited higher swimming speeds and shorter, more spatially constrained exploratory bursts. Conversely, the absence of visual input led to prolonged, dispersed exploration, particularly in triads. A data-driven stochastic model uncovered a nonlinear and noise-driven decision-making mechanism, demonstrating that visual input biases individuals towards conspecific proximity, whereas its absence facilitates extended exploration. Although our study focuses on singles, dyads and triads, these findings highlight a fundamental sensory–social interaction, suggesting that the number of individuals modulates the extent to which zebrafish rely on visual input for spatial navigation and decision-making. By integrating behavioural experiments with data-driven modelling, our results provide a promising framework for understanding how individual-level sensory processing scales to collective behaviour. Additionally, our results carry important ecological implications, as zebrafish frequently encounter fluctuating light conditions in the wild, which may directly impact fitness by influencing their ability to navigate, locate resources and assess risks [13–15].

In partial agreement with our expectations, we observed that zebrafish swimming activity was higher in the presence of visual input compared to the Dark condition. Under Bright illumination, fish exhibited higher linear and angular speeds regardless of the Number of Individuals (i.e. there was no interaction effect of Number of Individuals and Illumination with these variables). The absence of a significant effect of Number of Individuals on these speed metrics may indicate that these measures, while informative, do not fully capture the spatio-temporal dynamics of social interactions. Indeed, our analysis of exploratory behaviour quantified through interindividual distance revealed that visual cues influence the temporal and spatial dynamics of exploration. Under Bright illumination, fish engaged in shorter bursts of exploratory behaviour, with reduced amplitudes compared to the Dark condition. This suggests that visual input encourages closer proximity among fish, while its absence in the Dark leads to more dispersed and prolonged exploration.

Previous work has demonstrated that aggressive interactions in zebrafish dyads follow a structured temporal pattern, where behavioural transitions are not random but regulated by underlying social mechanisms [61]. Similarly, in [62], aggressive and mating behaviours were found to vary systematically with sex composition and time of day. Our findings extend this understanding by demonstrating that the effect of visual input on exploration dynamics is dependent on the number of individuals. While dyads exhibited no significant difference in exploration duration between Bright and Dark conditions, triads explored significantly longer in Dark than in Bright. Moreover, in Dark, triads engaged in more prolonged exploration episodes than dyads, despite both groups maintaining similar exploration amplitudes. This suggests that, in dyads, the absence of visual input does not drastically alter the balance between exploration and social interactions, potentially due to the more direct nature of dyadic interactions. In contrast, triads, where social coordination is distributed across multiple individuals, appear more sensitive to the loss of visual cues, leading to extended exploration episodes. Overall, the observed differences between dyads and triads suggest that they operate under distinct behavioural feedback mechanisms, further supporting the notion that group behaviour emerges from the interplay between sensory cues, social structuring and decision-making processes.

In addition, the observed reduction in swimming activity and the tendency for prolonged exploratory behaviour in Dark are consistent with previous research on fish behaviour in turbid waters [38,63] and with varying illumination [23]. Specifically, the authors found that guppies (*Poecilia reticulata*) were less active, formed smaller shoals, and were found to be more often alone in turbid than in clear waters [38]. Similarly, findings on three-spined sticklebacks [63] suggest that perceived predation risk increases in low-visibility environments, such as dark or turbid waters. This intensified perception of risk could stem from the unfamiliarity of the environment rather than an inherent association of darkness with predation threat.

On the other hand, in agreement with our second hypothesis, our analysis revealed a strong relationship between visual cues, quantified by opacity/optic flow and exploratory behaviour, measured through interindividual distance. Under Bright illumination, both opacity and optic flow were negatively correlated with the interindividual distance, indicating that higher visual field occupancy and greater relative motion between conspecifics lead to reduced spacing between fish. Notably, opacity and optic flow were higher in triads than in dyads, reflecting increased visual field occupancy due to the presence of more conspecifics. This observation can be explained by the concept of collective blind areas, where larger groups have an enhanced capacity to cover blind spots, resulting in a denser visual environment for each individual [46,64]. While the interindividual distance between dyads and triads did not significantly differ, the slope of the relationship between visual field occupancy and interindividual distance was steeper for dyads. This suggests that fish in dyads could rely more on the visual input of a single conspecific to regulate spacing, whereas in triads, visual information is distributed across multiple neighbours, reducing the influence of any single conspecific.

Interestingly, in Dark, the nature and strength of the relationships between visual cues (opacity and optic flow) and interindividual distance were significantly altered. In the absence of visual input, the slopes of these relationships became steeper, and for triads, the linear relationships were no longer statistically significant. This shift reinforces the validity of opacity and optic flow as proxies for visual input, as their explanatory power diminishes when vision is impaired. In addition, in Dark, fish exhibited increased interindividual distances, which may reflect a shift in sensory strategy, where reliance on non-visual modalities such as the lateral line and olfaction becomes more prominent [24,25,55]. The lateral line, in particular, is known to play a crucial role in detecting water flow and conspecific movement, and previous studies have shown that its disruption leads to increased interindividual distances in groups of firehead tetras [65]. Moreover, an alternative explanation for this adjustment in the Dark is that it reflects a cautious exploration strategy in a novel, unfamiliar environment, where fish prioritize risk avoidance in the face of uncertainty [63].

The data-driven modelling analysis uncovered a fundamental dynamic principle governing fish exploratory behaviour, revealing differences in the deterministic and stochastic components under varying illumination conditions. Under Bright illumination, the deterministic component, captured by the drift term, exhibited a biased potential function for both fish dyads and triads. This potential landscape favoured proximity to conspecifics, promoting intermittent transitions between cohesive group formation and far-distance exploration. Bistable dynamics are fundamental to biological systems, describing dynamics in bioelectric memory [66], gene regulation [67], ant colony decision-making [68] and human cognitive flexibility [69]. In zebrafish groups, Miller & Gerlai [6] documented transitions between schooling and shoaling, suggesting an intrinsic bistability modulated by habituation to the environment and group size.

The emergence of a bistable potential function in the drift term reveals the natural interplay between proximity to conspecifics and exploration, providing a quantitative lens through which to understand decision-making in animal collectives. The observation that the potential barrier height is reduced in the absence of visual input suggests that fish rely on visual feedback to sustain cohesive behaviour. In fact, in Dark, the bias of the potential function was significantly diminished, resulting in a more balanced landscape with a lower barrier between wells. This shift facilitated the transitions between close-proximity behaviour and distant exploration. This behaviour is further supported by the increased amplitude and prolonged duration of the exploration episodes.

Moreover, the stochastic component, represented by the diffusion term, followed a parabolic profile under both Illumination conditions. The noise was amplified at intermediate interindividual distances and minimized near the wells corresponding to close and far-distance states. This aligns with the intuition that when fish are at a central point of the arena, uncertainty in the decision-making process is maximal, requiring stronger noise to drive a choice. In contrast, noise diminishes as the fish approach their target state, reflecting higher certainty in their decision. Interestingly, this parabolic shape bears a conceptual resemblance to the entropy of a binary probabilistic system, such as a coin flip [70]. In this binary system, entropy is maximized when the probability of heads and tails is equal, representing maximal uncertainty. Similarly, the diffusion term indicates that maximal uncertainty occurs at intermediate interindividual distances, where the fish must break the indecision between remaining near conspecifics or engaging in exploration. Notably, the diffusion term does not exhibit noticeable differences between Bright and Dark conditions, maintaining a similar parabolic shape with only a slight shift in the centre for both fish dyads and triads. The diffusion term for fish triads has a higher amplitude compared to that of fish dyads, suggesting a greater influence of noise in the decision-making process for triadic groups. While a higher noise amplitude might initially suggest stronger random behaviour, it can also encapsulate the increased responsiveness emerging in triadic groups, where multiple social cues are processed simultaneously. This phenomenon aligns with social facilitation [13,15], whereby the presence of more conspecifics amplifies each individual’s propensity to switch states. In fact, supporting this notion, the results of visual input (opacity and optic flow) demonstrated a strong dependence on the Number of Individuals. An alternative perspective, consistent with [3], suggests that stochastic fluctuations can enhance, rather than disrupt, group cohesion. Similarly, the increased diffusion amplitude in triads may indicate a greater reliance on probabilistic social feedback.

Our study is not free of limitations, which call for future research in several directions. First and foremost, our analysis was restricted to fish groups of dyads and triads. While this choice was motivated by the rich interaction dynamics observed in small group sizes [27,35,36], larger groups may exhibit more complex behaviours. Future studies will investigate how group size beyond triads affects the interplay between visual feedback, exploration and collective decision-making. Second, our experimental conditions focused on two distinct illumination conditions: Bright and Dark. While this approach captures the extreme ends of visual input, natural aquatic environments often experience gradual changes in lighting due to factors like turbidity, depth and time of day [23]. Future work will investigate collective exploration under gradual or fluctuating illumination conditions. Third, our modelling approach, based on first-order stochastic differential equations, effectively captures group-level decision-making but does not account for individual-level dynamics. Future research will focus on developing agent-based models that incorporate individual visual cues and sensory processing, enabling a more granular understanding of the mechanisms driving collective exploration.

Although there are still various unexplored areas of future research, our study lays the groundwork for understanding collective exploratory behaviour, shedding light on the combined influence of Illumination and the Number of Individuals. Our findings underscore the fundamental role of visual input in driving collective dynamics that underpin group living, offering new perspectives on the mechanisms governing exploratory behaviour in animal collectives.

## Data Availability

Data and relevant code for this research work are stored in GitHub: [71] and have been archived within the Zenodo repository: [72]. Supplementary material is available online [73].
